# Exercise does not enhance aged bone's impaired response to artificial loading in C57Bl/6 mice

**DOI:** 10.1016/j.bone.2015.06.026

**Published:** 2015-12

**Authors:** Lee B. Meakin, Chinedu Udeh, Gabriel L. Galea, Lance E. Lanyon, Joanna S. Price

**Affiliations:** a School of Veterinary Sciences, University of Bristol, Bristol BS40 5DU, UK; b School of Clinical Sciences, University of Bristol, Bristol BS2 8DZ, UK

**Keywords:** Bone adaptation, Artificial loading, Strain, Exercise, Aging

## Abstract

Bones adapt their structure to their loading environment and so ensure that they become, and are maintained, sufficiently strong to withstand the loads to which they are habituated. The effectiveness of this process declines with age and bones become fragile fracturing with less force. This effect in humans also occurs in mice which experience age-related bone loss and reduced adaptation to loading. Exercise engenders many systemic and local muscular physiological responses as well as engendering local bone strain. To investigate whether these physiological responses influence bones' adaptive responses to mechanical strain we examined whether a period of treadmill exercise influenced the adaptive response to an associated period of artificial loading in young adult (17-week) and old (19-month) mice. After treadmill acclimatization, mice were exercised for 30 min three times per week for two weeks. Three hours after each exercise period, right tibiae were subjected to 40 cycles of non-invasive axial loading engendering peak strain of 2250 με. In both young and aged mice exercise increased cross-sectional muscle area and serum sclerostin concentration. In young mice it also increased serum IGF1. Exercise did not affect bone's adaptation to loading in any measured parameter in young or aged bone. These data demonstrate that a level of exercise sufficient to cause systemic changes in serum, and adaptive changes in local musculature, has no effect on bone's response to loading 3 h later. This study provides no support for the beneficial effects of exercise on bone in the elderly being mediated by systemic or local muscle-derived effects rather than local adaptation to altered mechanical strain.

## Highlights

• Aging impairs bones' response to artificial loading but not exercise.
• Treadmill exercise sufficient to invoke increases in muscle size and circulating IGF1 and sclerostin was selected.
• Exercise was combined with artificial loading sufficient to cause bone gain in young and aged mice.
• The systemic changes induced by exercise had no additional beneficial effect on the response to local artificial loading.
• This suggests the beneficial effects of exercise result from local changes in mechanical strain alone.

## 1 Introduction

Bone architecture adapts to changes in the mechanical strain engendered within it and by this means ensures that it becomes, and is maintained, sufficiently strong to withstand the loads to which it is subjected [1]. This negative feedback mechanism is widely known as the mechanostat. The mechanostat is primarily a local phenomenon with local changes in loading-engendered strain culminating in local bone adaptation [2]. However, bone architecture is also influenced by endocrine and paracrine changes which may have their effect directly through the mechanisms of the mechanostat or by influencing the context in which the mechanostat operates [3].

With age in humans, there is a decline in bone mass and architecture to the extent that bones become sufficiently fragile that low levels of trauma can lead to fracture [4]. In animal models, age has been shown to be associated with reduced function of the mechanostat as shown by reduced adaptive responses to loading compared with that in young adults [5], [6], [7], [8]. It has been recently suggested that this reduction in responsiveness does not occur at the stage where osteocytes respond to strain but at the subsequent stage where osteoblasts proliferate [8].

Although bones' adaptive response to loading is impaired with age, it is not eliminated in either humans or rodents. A recent meta-analysis of randomized controlled clinical trials concluded that exercise can increase bone mineral density in the lumbar spine and femoral neck, two regions predisposed to osteoporotic fracture [9]. A long-term exercise study in 14-month-old rats demonstrated that 16 weeks of exercise was sufficient to increase trabecular bone density [10] and cortical bone formation primarily on the periosteal surface [11]. Other studies in rats comparing the responses to resistance [12] and treadmill exercise [13] found increased bone formation in response to exercise in aged animals when directly compared with young adults.

However, when comparing results from studies using artificial loading and exercise in aged experimental animals, it is important to consider the differences between these models. Both provide a mechanical strain stimulus however, with artificial models this is controlled, well defined and unimodal. In comparison in exercise models it is usually neither measured nor controlled and includes diverse loading modalities [14], [15]. Exercise also invokes many additional physiological changes not engendered by artificial loading. These include increased blood flow and therefore tissue oxygenation [16], [17], local muscular contraction and the release of endocrine and local paracrine factors [18], [19], [20].

Changes in circulating concentrations of insulin-like growth factor (IGF)1 could explain the different results between exercise and artificial loading experiments. Although local concentrations of IGF1 have not been shown to change following artificial mechanical loading [21], [22], we have shown that loading increases the sensitivity of the IGF1-receptor to ambient IGF1 *in vitro* [23]. With age there is a decline in IGF1 [24], [25], [26], although treadmill exercise can lead to its increase in both young and aged animals [27], [28]. Therefore, an age-related decline in IGF1 could limit bone's response to artificial mechanical loading whereas its increase during exercise could rescue this impaired response. Changes in the expression of the Wnt antagonist sclerostin and/or glucocorticoids could also explain the impaired exercise-related response to loading in aged mice. Serum concentrations of sclerostin have been shown to be elevated in elderly humans with low bone mass [29], [30] and serum sclerostin is lowest in the quartile of women with the highest levels of physical activity [31]. Glucocorticoids are known to inhibit bone formation [33], [34], and corticosterone has been shown to be elevated in aged mice [32]. In humans that exercise [35] and mice with voluntary access to exercise wheels [36], serum glucocorticoid concentrations are reduced. Since multiple factors known to influence bone formation appear to be differentially regulated by exercise and aging, we here explored the hypothesis that altering systemic and muscular factors through exercise will enhance aged bone's diminished response to artificial loading.

## 2 Materials and methods

### 2.1 Animals

Young adult (17-week-old, n = 30) and aged (19-month-old, n = 35) female C57BL/6 mice were obtained from Charles River Inc. (Margate, UK). All mice were allowed free access to water and a maintenance diet containing 0.75% calcium (EURodent Diet 22%; PMI Nutrition International, LLC, Brentwood, MO, USA) in a 12-hour light/dark cycle, with room temperature at 21 ± 2 °C. All cages contained wood shavings, bedding and a cardboard tube for environmental enrichment. Mice were housed in groups of up to five animals [37]. All procedures complied with the UK Animals (Scientific Procedures) Act 1986 and were reviewed and approved by the University of Bristol ethics committee (Bristol, UK).

### 2.2 Exercise training

Mice were acclimatized to the treadmill three times over a one week period (see Supplementary Table 1). Subsequently, they were exercised for 30 min three times per week at 23 cm s^− 1^ for young adults and 18 cm s^− 1^ in aged mice. These speeds were selected as they have previously been shown to be the voluntary running speeds of this age of mice on an exercise wheel [38].

### 2.3 In vivo external mechanical loading

Right tibiae were subjected to external mechanical loading under isoflurane-induced anesthesia on three days per week for two weeks to investigate the effect of loading on bone (re)modeling. Loading was applied 2.5 h after exercise finished as exercise studies in humans indicate IGF1 mRNA concentrations are elevated at this time point [39], [40]. Left limbs were used as internal controls as previously validated [41], [42]. The protocol for non-invasively loading the mouse tibia has been reported previously [14], [43]. In brief, the flexed knee and ankle joints are positioned in concave cups and the upper cup containing the knee is attached to an actuator arm of a loading device and the lower cup to a dynamic load cell. The tibia is held in place by a 0.5 N continuous static pre-load. 40 cycles of dynamic load are superimposed with 10 s rest intervals between each cycle. The protocol for one cycle consists of loading to the target peak load, hold for 0.05 s at the peak load, and unloading back to the 0.5 N pre-load. From the strain gage data previously reported in these ages, strain and sex of mice [8] we applied 14 N to young adult and 11.1 N to aged mice giving a peak strain stimulus of 2250 με. The strain rate at this site was normalized to a maximum of 30,000 με s^− 1^ by loading and unloading at a speed of 500 N s^− 1^ and 393 N s^− 1^ in young adult and aged mice respectively.

### 2.4 Serum collection and analysis

Mice were killed by asphyxiation with CO_2_ and blood removed by direct cardiac puncture 3 h after the final period of exercise as studies document a peak increase in IGF1 mRNA concentrations at this time point[39], [40]. Serum was separated by centrifugation and stored at − 80 °C. Serum IGF1, IGF BP3 and sclerostin were measured using ELISA according to manufacturer's instructions (R&D Systems, Abingdon, UK). Serum corticosterone was assayed using a competitive radioimmunoassay as previously reported [44].

### 2.5 High-resolution μCT analysis

Following sacrifice, lower legs were stored in 70% ethanol and whole tibiae imaged using the SkyScan 1172 (Bruker, Kontich, Belgium) with a voxel size of 4.8 μm (110 μm^3^). The scanning, reconstruction and method of analysis has been previously reported [37], [45]. We evaluated the effect of age and sex on both tibiae and changes [(right − left) / left] ∗ 100 due to loading in bone volume fraction (BV/TV), trabecular thickness (Tb.Th), trabecular number (Tb.N) and trabecular pattern factor (Tb.Pf) in the trabecular region (0.25–0.75 mm distal to the proximal physis) and cortical bone area (Ct.Ar), total cross-sectional area inside the periosteal envelope (Tt.Ar), medullary area (Ma.Ar) and cortical thickness (Ct.Th) in the cortical site level demonstrating maximal bone formation following loading (37% from the proximal end) [41], according to ASBMR guidelines [46]. Cross-sectional muscle area (Mu.Ar) was also measured at the tibial mid-shaft using μCT.

### 2.6 Statistical analysis

Left control and right loaded limbs and change in bodyweight were compared using a paired t-test. The combined effect of age and exercise on bodyweight, tibial length, serum, bone and muscle parameters and on the percentage change in bone mass and architecture due to mechanical loading were assessed using a two-way ANOVA with post-hoc Bonferroni correction where significant interactions were detected. All data is reported as mean ± SEM. Significance was set at p < 0.05. Analyses were performed using GraphPad Prism (version 6.0, GraphPad Software, La Jolla, CA, USA).

## 3 Results

### 3.1 Bodyweight and tibial length

There was no significant difference in bodyweight between control and exercise groups within young adult or aged mice at the start or end of the study period (p > 0.05, Table 1). Aged mice were significantly heavier than young adults (p < 0.001). Both groups of aged mice lost similar bodyweight during the study period (control − 4.4%, exercise − 3.5%, p < 0.001). The tibias of aged mice were significantly longer than in young adults (3.8%, p < 0.001). There was no difference in length between left control and right loaded tibiae in any group (p > 0.05). All data is summarized in Table 1.

### 3.2 The effect of age and exercise on serum parameters

As expected from previously published data, exercise increased serum IGF1 concentration in young mice (15%, p = 0.02 by unpaired t-test) although this increase was no longer significant when the data was analyzed collectively in the two-way ANOVA. There was no significant change in IGFI concentration associated with age (p > 0.05). Since the majority of circulating IGF1 is bound to IGF BP3, this was also measured. Overall there was a significant decrease in IGF BP3 with age (p < 0.001) with no overall effect of exercise (p > 0.05).

There was a significant increase in corticosterone in aged compared to young animals (p = 0.03). This elevation was predominantly in the non-exercised groups (128% increase) although the interaction did not reach statistical significance.

Serum sclerostin was overall significantly increased with exercise in both young and aged mice (p = 0.04). In contrast, there was no effect of age alone (p > 0.05).

### 3.3 The effect of exercise on bone mass and architecture and muscle area

As described in the Materials and methods, we aimed to select a level of exercise that was not itself osteogenic but was sufficient to induce some of the systemic changes associated with exercise which were likely to influence bones' adaptive response to loading. Therefore, as expected, there was no significant effect of exercise alone on any calculated parameter in trabecular or cortical bone as assessed by comparing left tibiae from mice which were subjected to exercise to those which were not (p > 0.05, data not shown).

In contrast to the lack of effect of exercise on bone mass and architecture, there was a significant overall effect of exercise on muscle area (p = 0.04). There was no significant effect of age on muscle area (p > 0.05).

### 3.4 The effect of mechanical loading on bone mass and architecture

**Fig. 1:**
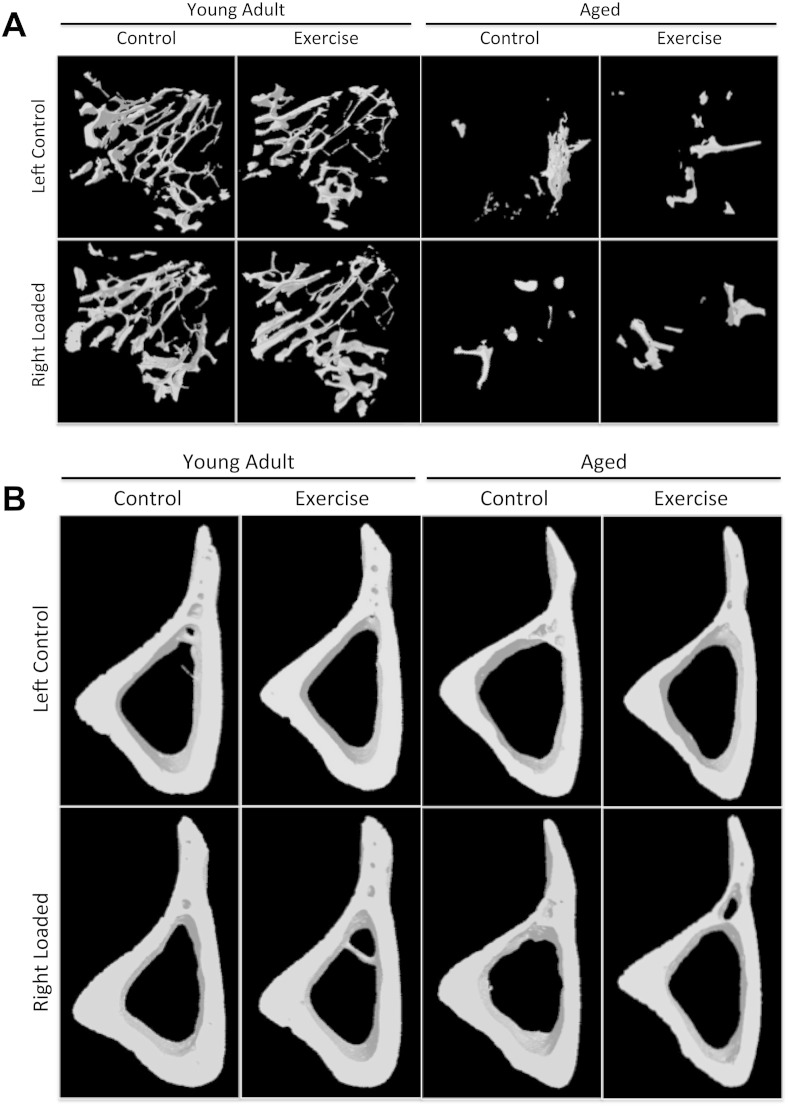
The effect of artificial loading and exercise on trabecular bone mass and architecture in young adult and aged female mice. Representative 3D reconstructions showing the effect of loading and exercise on the region of (A) trabecular bone and (B) cortical bone analyzed by μCT.

The effect of loading on bone mass and architecture using the axial tibial loading model in these ages of mice has previously been described [8]. In the current study, we used a sub-maximal strain stimulus of 2250 με so that any additional effect of exercise could be appreciated. Loading to this magnitude of peak strain produced changes in bone mass and architecture consistent with previous studies (Table 1) [8], [47]. In trabecular bone, there was a loading related increase in BV/TV in young mice (59.6% ± 4.3, p < 0.001) with a smaller but still significant increase in BV/TV in aged mice (28.6% ± 15.3, p = 0.03). This increase was primarily due to an increase in Tb.Th in both ages of mice (young 25.6% ± 2.7, p < 0.001; aged 29.8% ± 7.2, p < 0.001). Tb.N also significantly increased with loading in young mice (28.1% ± 4.8, p < 0.001), although no significant change was observed in aged mice (36.1% ± 28.6, p > 0.05). Tb.Pf, a measure of bone connectivity, was significantly decreased by mechanical loading in young mice (− 23.3% ± 2.7, p < 0.001) suggesting an increase in connectivity. In contrast there was no significant change in Tb.Pf in aged mice due to loading (− 7.75% ± 8.14, p > 0.05, Fig. 1A).

In cortical bone, there was a significant loading-related increase in Ct.Ar in both ages of mice (young 14.8% ± 1.2, p < 0.001; aged 10.1% ± 1.7, p < 0.001). This was due to a significant increase in Tt.Ar in young mice (7.69% ± 1.2, p < 0.001) with no change in Tt.Ar in aged mice (1.36% ± 1.4, p > 0.05). In contrast there was no significant change in Ma.Ar in young mice (− 1.84% ± 2.2, p > 0.05) but a significant decrease in aged mice (− 7.02% ± 3.2, p = 0.03). Ct.Th was significantly increased by loading in both young adult (13.0% ± 1.2, p < 0.001) and aged mice (9.31% ± 2.8, p < 0.001, Fig. 1B).

### 3.5 The effect of age and exercise on bone's response to mechanical loading

We next investigated whether there were any differences in bones' adaptive response to loading due to age or exercise. This comparison showed no additional effect of exercise on bone's response to artificial loading for any measured parameter in trabecular or cortical bone. The only significant finding was that aging reduced the loading-related increase in Tt.Ar (p = 0.002).

## 4 Discussion

The results of this study demonstrate that levels of exercise in young and aged mice sufficient to increase local muscle area and engender measurable changes in serum concentrations of IGF1 and sclerostin had no measurable beneficial effect on the adaptive responses of cortical or trabecular bone to artificial mechanical loading imposed 3 h later.

The exercise protocol used was intended to engender systemic changes in circulating hormones and muscle mass but not itself engender any significant osteogenic response. As expected from previous studies [28], [39], [40], exercise increased IGF1 in young mice. However, no such increase was observed in aged mice. This contrasts with the results from a study by Wills et al. [27]. This difference is likely to reflect differences in exercise regimens. In our study there were no overall changes in total IGF1 with age. However, there was an age-related decrease in circulating IGF BP3 which mirrors age-related changes in humans [48], [49]. This would suggest an increase in free IGF1 since the bound fraction is likely to be lower. This contrasts with the decline in IGF-signaling reported with age in humans [25].

As expected from previous human and rodent studies, corticosterone was elevated in aged mice compared to young controls [32], [50]. Interestingly, exercise appeared to reduce this elevation to a level no different from young control and exercised mice. This is consistent with studies in young mice but to our knowledge has not previously been investigated in aged mice [36]. Finally, we examined the effect of exercise and age on serum sclerostin. There was no overall effect of age but a significant increase with exercise. This has previously been documented in humans 1 h after exercise [51] although chronic levels of increased activity were associated with a lower serum sclerostin concentration [31]. The role of the osteocyte-specific protein sclerostin in the serum is still under debate so the significance of this finding is currently unclear.

In addition to the perturbations in serum hormones, exercise also caused a significant increase in muscle cross-sectional area at the mid-shaft of the tibia. This is a further positive indication that the level of exercise was sufficient to induce both local and systemic effects. There has been recent interest in the interaction between bone and muscle so data showing that a level of exercise sufficient to cause an increase in muscle cross-sectional area has no effect on local loading-related bone gain suggests that the two are not causally related. This is consistent with the finding that the age of mice used in this study had measurable age-related deterioration in bone mass and architecture but no change in muscle area compared to young adult mice. One of the potential beneficial effects of exercise which could not be explored in this animal model was that of coordination. It is well accepted in human studies that exercise will improve neuromuscular control to reduce the risk of falls and therefore fragility fractures. Thus our study in no way suggests that exercise is not valuable in preventing fractures in old people only that we see no evidence that there is a direct relationship between the factors associated with increasing or maintaining muscle size with the responses to mechanical loading associated with regulating bone mass and architecture.

The changes in bone mass and architecture in response to loading shown in our current study are consistent with data previously published by ourselves [8] and others [5], [6], [7], [52]. It is now apparent that despite similar increases in trabecular thickness with loading in aged compared with young adult mice, overall there is a smaller, albeit still significant, increase in overall BV/TV. This can be accounted for by an age-related abrogation of the positive effects of loading on trabecular number and connectivity observed in young adult mice. In cortical bone, it became more apparent that aging, as well as impairing the magnitude of bones' adaptive response to loading, also changed the sites of bone formation. In young adults bone formation was exclusively periosteal while in aged mice it was endosteal. This is consistent with previous histomorphometric studies [53].

The limitations of this study include the length of time selected for the exercise period. A more protracted period of exercise could have lead to a greater increase in bone mass and architecture in both age groups of mice. However, maximizing this response was not the primary purpose of our study. It is possible that the timing of the exercise was such that it did not engender any beneficial effects on bones' adaptability and that another timing or another exercise regimen would have different effects. Although the level of exercise selected for this study was sufficient to cause an increase in muscle area at the tibial midshaft, we did not measure muscle mass or strength. There were also no longitudinal measurements of the physiological responses to exercise. In addition it is possible that artificial mechanical loading itself could induce systemic changes in addition to those induced by exercise. Although in our view this is unlikely to be significant and all mice underwent artificial loading of one limb for consistency, we cannot exclude the possibility that loading could have influenced the serum analyses in this study.

We had hypothesized that the systemic and local muscular physiological changes induced by exercise would enhance bone's adaptive response to loading, perhaps elevating it in aged mice to a level similar to that observed in young mice. In fact the data presented here suggest no beneficial effects of exercise on bones' adaptive to strain. Our conclusion is therefore that the frequently reported beneficial effects of exercise on bone mass and architecture in aged animals are likely to reflect bones' adaptation to changes in local mechanical strain alone rather than any additional physiological response.

The following are the supplementary data related to this article.

Supplementary Table 1 Speed and duration of treadmill exercise during the acclimatization and study periods.

## Authors' roles

Study design: LBM, GLG, LEL and JSP. Study conduct and data collection: LBM and CU. Data interpretation: LBM, CU, GLG, LEL and JSP. Drafting manuscript: LBM and LEL. Revising manuscript content: GLG, LEL and JSP. Approving final version of the manuscript: LBM, CU, GLG, LEL and JSP. LBM takes responsibility for the integrity of the data analysis.

## Conflict of interest

All authors declare that they have no conflict of interest.

## Figures and Tables

**Table 1:** The effect of age, exercise and loading on bodyweight, tibial length, trabecular and cortical bone parameters and the results of serum analyses. Bones were analyzed using high-resolution μCT. Serum was analyzed using ELISA (IGF1, IGF BP3, sclerostin) or RIA (corticosterone). Data represented as mean ± SEM (young control and aged n = 15; young exercise n = 9). ^a^p < 0.05, ^b^p < 0.01, ^c^p < 0.001 comparing change in bodyweight during the study period or left control with right loaded limbs using paired t-test. Results of a two-way ANOVA to assess the effect of age, exercise and their interaction on bodyweight, tibial length, muscle area, serum parameters and the loading-related percentage change in bone parameters are also presented; significant results (p < 0.05) are in bold.

Age	Young adult		Aged		Two-way ANOVA p-values		
Exercise group	Control	Exercise	Control	Exercise	Age	Exercise	Interaction
Bodyweight (g)							
Start	21.8 ± 0.2	21.7 ± 0.2	27.7 ± 0.8	28.6 ± 0.5	**< 0.001**	0.41	0.47
End	22.0 ± 0.2	21.9 ± 0.3	**26.4 ± 0.7^c^**	**27.6 ± 0.5^c^**	**< 0.001**	0.29	0.29
Tibial length (mm)							
Left control	17.7 ± 0.05	17.7 ± 0.05	18.3 ± 0.10	18.5 ± 0.04	**< 0.001**	0.076	0.066
Right loaded	17.8 ± 0.06	17.7 ± 0.04	18.3 ± 0.09	18.5 ± 0.05	**< 0.001**	0.44	0.31
Muscle area (mm^2^)	0.0402 ± 0.0008	0.0409 ± 0.0005	0.0387 ± 0.0007	0.0413 ± 0.0008	0.54	**0.042**	0.22
Trabecular bone							
BV/TV (%)							
Left control	6.56 ± 0.20	6.60 ± 0.38	1.41 ± 0.18	1.49 ± 0.19	0.71	0.64	0.089
Right loaded	**10.39 ± 0.27^c^**	**9.42 ± 0.18^c^**	**1.92 ± 0.27^a^**	**2.03 ± 0.31^a^**
Tb.Th (mm)							
Left control	0.0464 ± 0.0008	0.0478 ± 0.0010	0.0522 ± 0.0022	0.0541 ± 0.0031	0.64	0.39	0.88
Right loaded	**0.0580 ± 0.0007^c^**	**0.0575 ± 0.0013^c^**	**0.0667 ± 0.0033^c^**	**0.0679 ± 0.0046^a^**
Tb.N (mm^− 1^)							
Left control	1.423 ± 0.054	1.379 ± 0.067	0.272 ± 0.036	0.263 ± 0.032	0.37	0.61	1.00
Right loaded	**1.797 ± 0.055^a^**	**1.643 ± 0.041^b^**	0.291 ± 0.039	0.293 ± 0.047
Tb.Pf (mm^− 1^)							
Left control	31.8 ± 0.49	32.2 ± 1.16	39.3 ± 2.56	40.3 ± 4.46	0.20	0.63	0.21
Right loaded	**24.3 ± 0.78^c^**	**26.0 ± 0.63^c^**	34.1 ± 2.79	**31.1 ± 4.06^a^**
Cortical bone							
Ct.Ar (mm^2^)							
Left control	0.704 ± 0.0067	0.699 ± 0.0094	0.535 ± 0.0106	0.533 ± 0.0107	0.31	0.44	0.18
Right loaded	**0.808 ± 0.0089^c^**	**0.793 ± 0.0081^c^**	**0.596 ± 0.0143^c^**	**0.613 ± 0.0121^c^**
Tt.Ar (mm^2^)							
Left control	1.21 ± 0.013	1.22 ± 0.027	1.09 ± 0.022	1.10 ± 0.024	**0.002**	0.54	0.38
Right loaded	**1.31 ± 0.013^c^**	**1.28 ± 0.013^a^**	1.12 ± 0.022	1.14 ± 0.018
Ma.Ar (mm^2^)							
Left control	0.510 ± 0.0097	0.519 ± 0.021	0.567 ± 0.022	0.562 ± 0.020	0.16	0.93	0.76
Right loaded	0.498 ± 0.0084	0.491 ± 0.012	**0.522 ± 0.017^a^**	0.531 ± 0.019
Serum							
IGF1 (ng/ml)	308 ± 12	354 ± 12	357 ± 32	347 ± 17	0.30	0.37	0.17
IGF BP3 (ng/ml)	486 ± 22	534 ± 40	387 ± 24	427 ± 23	**< 0.001**	0.16	0.95
Corticosterone (ng/ml)	133 ± 26	145 ± 46	305 ± 69	172 ± 31	**0.030**	0.17	0.11
Sclerostin (pg/ml)	95.8 ± 5.6	106.3 ± 3.9	93.9 ± 4.8	110.2 ± 5.6	0.85	**0.038**	0.66
