# Supplementary material for: Exercise does not enhance aged bone's impaired response to artificial loading in C57Bl/6 mice
Source: Bone. 2015 Dec;81:47–52. doi: 10.1016/j.bone.2015.06.026 (PMC4652609; doi:10.1016/j.bone.2015.06.026)
Supplement: Supplementary Table 1 — Speed and duration of treadmill exercise during the acclimatization and study periods. [file mmc1.docx]

Supplementary Table 1

| Day number | Young Adult Speed (cms^-1^) | Aged Speed (cms^-1^) | Time (min) |
| --- | --- | --- | --- |
| 1 | 8 | 8 | 5 |
| 3 | 12 | 10 | 10 |
| 5 | 16 | 12 | 20 |
| 8-22 | 23 | 18 | 30 |

Supplementary Table 1: Speed and duration of treadmill exercise during the acclimatization and study periods.
